# Does vibrotactile biofeedback for postural control interfere with cognitive processes?

**DOI:** 10.1186/s12984-024-01476-w

**Published:** 2024-10-18

**Authors:** Katrin H. Schulleri, Farbod Feizian, Martina Steinböck, Dongheui Lee, Leif Johannsen

**Affiliations:** 1 Human-centered Assistive Robotics, Technical University of Munich, Karlstraße, 80333 Munich, Bavaria Germany; 2 Department Health and Sport Sciences, Technical University of Munich, Georg-Brauchle-Ring, 80992 Munich, Bavaria Germany; 3 Department of Neurology, Research Group, Schoen Clinic Bad Aibling, Kolbermoorer Straße, 83043 Bad Aibling, Bavaria Germany; 4 Autonomous Systems, Technische Universität Wien, Gusshausstrasse, 1040 Vienna, Austria; 5 Institute of Robotics and Mechatronics, German Aerospace Center (DLR), Münchener Straße, 82234 Wessling, Bavaria Germany; 6 Institute of Psychology, RWTH Aachen University, Jaegerstrasse 17/19, 52066 Aachen, Germany

**Keywords:** Cognitive demand, Cognitive interference, Biofeedback, Balance control

## Abstract

**Background:**

Directional vibrotactile biofeedback for balance control can be instructed in the form of *Repulsive* (to move in the opposite direction of vibrations) or *Attractive* (to move in the direction of vibrations) stimulus encodings. However, which of these encodings is less cognitively demanding and poses less interference with high-level cognitive processes of conflict resolution remains unresolved.

**Methods:**

In two between-subject studies with 30 (16 females) and 35 (23 females) healthy young adults, respectively, we investigated the cognitive load of *Attractive* and *Repulsive* vibrotactile biofeedback on 1) working memory (Study I) and 2) cognitive conflict resolution (Study II). Both studies also examined the effectiveness of both feedback stimulus encodings on balance control during quiet standing with eyes closed.

**Results:**

Both *Attractive* and *Repulsive* vibrotactile biofeedback increased balance stability (reduced trunk sway variability) in both the working memory and the conflict resolution study (Study I and II, respectively) with a greater increase of stability for the *Repulsive* encoding during multitasking demanding cognitive conflict resolution (Study II). Cognitive costs, measured in terms of the Linear Integrated Speed-Accuracy Score (LISAS), were greater for the *Attractive* encoding during multitasking with working memory demands. When cognitive conflict resolution was required as a secondary cognitive task, both stimulus encodings increased cognitive costs equally.

**Conclusions:**

The effects of instructed *Repulsive* and *Attractive* stimulus encodings for the response-related interpretation of vibrotactile biofeedback of body sway were contrasted with respect to cognitive processing demands and balance stabilisation benefits. Both encodings improved balance stability but at certain cognitive costs. Regarding interference with specific high-level cognitive processes, however, a distinction has to be made between both encodings. Repulsive feedback encoding seems to cause less cognitive costs on working memory load and slightly greater stabilisation when cognitive conflict resolution is required. These results are discussed in the context of the known benefits of avoidance actions on cognitive control.

**Supplementary Information:**

The online version contains supplementary material available at 10.1186/s12984-024-01476-w.

## Background

Maintaining functional independence in daily life is essential for avoiding high personal, societal and healthcare costs, particularly in a growing older population [1]. Limitations in functional independence are reflected in impaired instrumental (e.g. using the phone, shopping, housecleaning, using public transportation, etc.) and functional activities of daily life (e.g. getting out and into bed, bathing, dressing, using the toilet, etc.), as well as in general mobility, physical activity and cognitive tasks [1]. Maintaining overall functional independence means the capability to perform any activities of life successfully. A necessary requirement for this is effective and efficient control of body balance, e.g. in terms of the utilisation of sensorimotor and cognitive processing resources [2, 3]. When performing two tasks simultaneously, for example, as in cognitive-postural multitasking, performance costs have been reported in the postural task domain [4, 5], in the cognitive domain [6], and both domains [7]. Which of these two tasks is more affected by any multitasking costs depends on their relative task prioritisation [8] influenced by contextual factors such as the difficulties of both the cognitive and postural tasks or the perceived level of postural threat, meaning an expected injury of certain severity should loss of balance and a fall occur [9]. A performance deterioration in a secondary cognitive task coinciding with an increase in balance stability has been attributed to a ’posture-first’ control strategy [10, 11], which assumes that participants prioritise postural performance, meaning the distribution or allocation of attentional resources in favour of the postural task, to prevent a fall [12]. In contrast to the general assumption of flexible attentional resource allocation causing multitasking interference stands the theory of a so-called central processing ’bottleneck’, which predicts response delays in a secondary task due to capacity limitations in response selection processes that are shared by the primary and secondary tasks [10, 13]. Thus, structural bottlenecks in response selection may lead to process interference [14] that could impact both cognitive and postural performance [12–18]. Cognitive-balance interference might increase the more complex and challenging task pairings become, either by the inclusion of a more difficult cognitive task and/or a more difficult balancing task [15, 19]. On the other hand and somewhat counterintuitively, however, several studies have shown that an easy secondary cognitive task can facilitate postural control to a certain extent [19–22], particularly when performing a more challenging balance task, such as standing on a sway-referencing surface with eyes closed for older adults [21]. The benefit of an easy secondary cognitive task was observed in both young and older adults and explained by a less disruptive top-down attentional focus on lower-level processes involved in sensorimotor control of balance [15, 17, 19–21, 23]. The so-called ”constrained action hypothesis” [24–26] suggests that distraction of the attentional focus by an easy cognitive task allows increased ”automaticity” of the sensorimotor system and, thereby, the release of more efficient postural control. In terms of sensory augmentation strategies, the control of body balance is also facilitated by different types of biofeedback that have been proven to be effective mainly in static balance [27], including auditory [28], electro-tactile [29], vibrotactile [30, 31], and multimodal [32, 33] types of feedback. Nonetheless, it seems that tactile stimuli applied to the trunk are preferred over other modalities, as they may interfere less with other sensory cues required during activities of daily life [27, 34–36]. In addition to the various effects of the location where feedback is applied, [37], different ways to encode directional vibrotactile feedback also need to be taken into consideration. Directional vibrotactile biofeedback has been demonstrated to enhance postural control during upright standing in various groups, including young and older healthy individuals, as well as patients with vestibular impairments (e.g., after stroke, Parkinson’s disease, or unilateral vestibular hypofunction and loss) [38–42]. Commonly, feedback is provided as soon as postural instability is detected, often based on trunk movements exceeding a predefined threshold [35, 43–45]. Thus, within a predefined zone representing a stable and safe state of balance, no feedback is given (”dead zone”). In many studies, users were instructed to move in the direction opposite to the sites of vibration (*Repulsive* feedback encoding) [35, 43–45], and some studies instructed users to move in the same direction to the sites of vibration (*Attractive* feedback encoding) [46–50]. Thus, *Repulsive* encoding could be interpreted as an ’avoidance’ signal indicating that the individuals have deviated from a stable postural position and need to move in the opposite direction [43, 45, 47, 49, 51, 52]. In a complementary fashion, *Attractive* encoding implies that users need to move in the direction of vibrations (’approach’ signal) to increase the stability of their postural state [46, 47, 49, 50]. Studies comparing these two instructed encodings suggest that both are effective in reducing body sway and increasing the percentage of time spent within the dead zone (stable area: feedback inactive). However, a *Repulsive* encoding was rated as subjectively more intuitive concerning the perceived familiarity and learning effort [47, 49].

Nevertheless, what is also unknown to date is the impact of vibrotactile biofeedback on sensorimotor control of balance in terms of interference costs with diverse cognitive processes (e.g. attentional control and working memory, executive functions, etc.) [53, 54]. Task representation in working memory and cognitive conflict resolution may play an important role in the interpretation of vibratory biofeedback. Any lack of knowledge regarding cognitive involvement is lamentable as any degree of cognitive interference would affect the usability and applicability of vibrotactile biofeedback during activities of daily life, particularly during more complex multitasking situations.

Cognitive demand can be assessed by performing multiple tasks simultaneously (multitasking), e.g. balance and cognitive tasks, each with and without vibrotactile feedback. During balance-cognitive multitasking, vibrotactile biofeedback improves balance control (reduces trunk sway variability and increases time within the dead zone), but it negatively affects cognitive task performance (i.e. response time) [41, 55, 56]. In these studies, the balance task, including the responses to the vibrotactile feedback, was prioritised. However, these studies used a *Repulsive* encoding only. While some studies found the *Repulsive* encoding to be subjectively more intuitive [47, 49], others found individuals to move involuntarily in the direction of vibration when vibrotactile feedback was uncoupled to human body sway and uninstructed [56, 57]. Consequently, it remains still unclear to what extent the two feedback encodings (*Repulsive* vs *Attractive*) impose different cognitive loads but also whether they show different patterns of interference with diverse cognitive processes. For example, if one of the two instructed stimulus encodings adhered to a more ’natural’, congruent directional coupling between sites of vibration and postural response, then the other may be termed incongruent and potentially in need of internal (cognitive) conflict resolution during response selection. To what extent does the *Attractive* or *Repulsive* vibrotactile biofeedback introduce an additional conflict that needs to be resolved?

In this study, we, therefore, aimed to investigate any multitasking interference evoked by *Attractive* and *Repulsive* vibrotactile biofeedback between balance control and cognitive processes, such as working memory (Study I) and cognitive conflict resolution (Study II). We expected both *Attractive* and *Repulsive* stimulus encoding to reduce trunk tilt variability and increase the time spent in the dead zone during quiet standing. Moreover, we expected increases in cognitive load with vibrotactile feedback, at least for the *Repulsive* encoding, as well as further increases with greater cognitive task difficulty and complexity. Finally, we assumed that one of the two stimulus encodings might be more demanding in terms of an intrinsic response conflict, for example, by the requirement to inhibit a ”natural” response tendency, and that this demand for conflict resolution would result in a greater incongruency effect in the Simon task for that particular encoding.

## Methods

Ethical approval declarations: The studies were conducted in accordance with the ethical code of conduct of the Helsinki Declaration and approved by the ethical committee of the Technical University of Munich (2019-248 1-S-SR). All participants gave written informed consent.

### Participants

Sample sizes in this work were based on previous studies [47, 49] that found an increased subjectively perceived familiarity and effort of learning for the *Repulsive* encoding in thirty young adults in a between-subject study (15 per group) and eight older adults in a within-subject study. The study of Tannert et al. [49] also showed that body sway was significantly decreased, and the percentage of time spent within the deadzone was significantly increased with vibrotactile biofeedback across both groups. Further, we considered the study of Haggerty et al. [55] that observed an increase in response time to a choice reaction time task due to repulsive vibrotactile biofeedback during balance-cognitive multitasking in ten older adults. In addition, they also found a significantly reduced body sway and an increased percentage of time spent in the deadzone, even during multitasking. Accounting for a smaller effect of multitasking in younger adults, including an auditory cognitive task [41, 58] and accounting for dropouts we recruited 32 (Study I: working memory) and 42 (Study II: conflict resolution) young adults between the ages of 18 and 35 years. In these between-subject studies with 16 participants in each encoding group in Study I and 21 in Study II, we investigated the effects of vibrotactile biofeedback encodings with additional 1) working memory demands (Study I) and 2) demands to resolve cognitive conflict (Study II), respectively.

Individuals were included to participate in our studies if they did not report any neurological, musculoskeletal, vestibular, or other diseases that could influence their independent standing and hearing abilities. Participants were assigned by covariate adaptive randomisation to one of the two equal-sized groups, balanced by gender. Due to the dropout of two participants in Study I, 15 (7 males, 8 females) participants per group were included for statistical analysis. In Study II, also two participants dropped out and five had to be excluded due to missing data. Thus, final analysis was performed with 18 (6 males and 12 females) and 17 (8 males and 12 females) individuals in the *Attractive* and *Repulsive* group, respectively. Individuals did not differ between groups in either of their baseline measurements, such as age, anthropometry, dead zone threshold, vibrotactile intensity and baseline sway variability (Single RMS) (Table B1).

### Instrumentation

To provide vibrotactile biofeedback, we used a haptic vest similar to the one used in our previous work [49]. It consists of two vibrotactile motors in the front and at the back (10 mm vibration motor 310–122; Precision Microdrives Inc.). Feedback was given as soon as the trunk tilt angle exceeded a threshold based on individual baseline trunk motion. The trunk angle was assessed by an inertial measurement unit (IMU) (MTW Awinda by Xsens). Further, to assess not only trunk motion but also the underlying control effort based on the centre of pressure, additionally, a force plate (AMTI, six-axis) was used in the working memory study. The sampling frequency was set to 100 Hz for both IMU and force plate. Finally, for the auditory cognitive tasks used in both studies, we used push buttons connected via an Arduino to the computer for the participant’s response. The button was fixated at participant’s index finger to be pressed by the thumb. The auditory stimuli were presented via a $360^{\circ }$ speaker (Jabra Speak 510) in the working memory study (Study I) and two speakers (Pioneer DJ, DM-40BT-W active monitor Speaker, AlphaTheta EMEA, JPN) in the conflict resolution study (Study II).

### Experimental procedure

For initial preparation, we followed the procedure as described by Tannert et al. [49]. This included the assessment of the just noticeable vibrotactile threshold by the methods of limits [59] and the assessment of baseline sway during upright narrow (2.5cm inter-foot distance) normal bipedal stance with eyes closed for 3 times of 35 s duration. The vibrotactile intensity was defined in both studies as 130% and 120% of the mean vibrotactile threshold of the two back motor locations [49]. Similarly, as in the previous study [49], the average vibrotactile threshold of the two back motors only was determined and used in the study, as the tactile sensitivity at the back compared to the front of the trunk has been shown to be lower [60]. For the first study, to trigger vibrotactile biofeedback, the threshold was defined as 1.2 times the absolute mean tilt angle assessed during baseline sway measurements for AP and ML (body sway-based threshold), respectively. In the second study, instead of a body sway-based threshold, we implemented a Limits-of-Stability-based threshold, as in a previous study, we have found this to better capture postural instability [61].

#### Study I—working memory

Participants stood in an upright and relaxed narrow Semi-Tandem stance (2.5cm inter-foot distance, toes of the rear foot at height of midfoot of the front foot) with eyes closed, their dominant foot (preferred foot to kick a ball) in front and their arms relaxed on their sides. First, familiarisation trials were performed to ensure how the vest works. Familiarisation trials were repeated until the participant felt comfortable. Based on the group they were assigned to, individuals were either instructed to move in the direction of vibrations (*Attractive*) or to move in the opposite direction of vibrations (*Repulsive*). To investigate the effect of these vibrotactile feedback encodings on cognitive load, we provided two auditory balance-cognitive multitasking conditions with different levels of difficulty/complexity. In the simple multitask, which was a choice reaction time task (CRT) [19, 58], participants heard a set of random numbers from 1 to 9 and were asked to press the right push-button when they heard either 1, 2 or 3 and press the left for any other number (Fig. A1). For the second cognitive task, we used the more difficult and more complex digit 2-back working memory task (2back) [19]. In this task, the users had to press the right push button if the number they heard was similar to the numbers they heard two steps back. Otherwise, they had to press the left push button. The cognitive tasks were implemented in MATLAB using psychtoolbox [62]. Before starting the measurements, participants performed one familiarisation trial for each cognitive task. Each cognitive task was conducted during quiet standing with (F) and without (nF) feedback. Moreover, the single-task quiet standing was also conducted with feedback. These five conditions (each 5 trials x 35 s + 30 s between trials) were conducted in a block-randomised order.

#### Study II—conflict resolution

Participants also stood in an upright and relaxed narrow (2.5cm inter-foot distance) stance with eyes closed; however, this time, in a parallel stance (Romberg stance). Also, in this study, individuals were first familiarised with the vest during 3 trials of each 35 s, which was followed by another three baseline measurements with feedback. Like in the first study, individuals were instructed according to the encoding group they were randomly assigned to. To investigate differences in processing interferences between the *Attractive* and *Repulsive* encodings, individuals performed an auditory Simon task [63] during quiet standing with eyes closed with and without feedback (Feedback (F)/no-Feedback (nF)) in a randomised order. All participants were equally divided into two different pitch condition groups through simple randomisation and instructed to either:

- Press the right key when the tone is high and press the left key when the tone is low (Pitch condition group 1, PC1), or
- Press the left key when the tone is high and the right key when the tone is low (Pitch condition group 2, PC2).

The auditory stimuli were present on the right and left ear so that the stimulus location was congruent or incongruent with the response identity of the stimulus. For example, when being instructed to press the right button for high pitch hearing the high pitch on the left ear would present an incongruent target. In addition, stimuli were presented to both ears at the same time as a neutral condition (Fig. A2). Each congruency condition was assessed by 26 trials each per block in randomised order. Ten blocks were performed in total in a block randomised order, five blocks with and five without vibrotactile feedback, consisting of 72 trials each. A single block took about 4.5 min to complete. Consequently, 720 trials were performed per participant with a minimum total testing duration of approximately 60 min (including a 1–2-minute break after each block and a 5-minute break after the fifth or sixth block). If the participants needed more time to rest, a more extended break was given. Before the experiment started, participants were allowed to test the push buttons and were presented three “high” and three “low” tones to prepare for the task. After completing all experimental trials, participants were asked to evaluate their satisfaction with the vest as an assistive technology (QUEST: Quebec User Evaluation of Satisfaction with Assistive Technology).

### Data processing and statistical analysis

Data were post-processed with MATLAB, with the exclusion of the first and last 2.5 s of a trial, and Butterworth low-pass filtered with a 5 Hz cut-off frequency to smooth the force plate data. To evaluate cognitive load based on cognitive task performance, we extracted the Linear Integrated Speed-Accuracy Score (LISAS) [58]. For the postural domain, we computed the following dependent variables as a measure of trunk motion and balance control performance with respect to the feedback, respectively:

- trunk tilt variability (RMS of tilt angle (°) at L5 lumbar vertebra) in AP (roll) and ML (pitch)
- Time-in-DZ in AP (roll) and ML (pitch): Percentage of time spent in dead zone based on the tilt angles

Instead of differentiating for direction, the second study investigated the RMS and Time-in-DZ based on the total tilt angle (TT).

For statistical analysis, we computed mixed model ANOVAs for cognitive load and balance control parameters with Group (*Attractive* vs. *Repulsive*) as a between-subject factor and Task (CRT/two-back) or (Single/Simon task), as well as Feedback (F/nF) and Direction (AP vs. ML) as within-subject factors. Further, for the feedback condition of the multitasking study, separate mixed model ANOVAs were computed to investigate the multitasking cost compared to the single task with feedback. In case of violation of sphericity, Greenhouse-Geisser (GG) correction was reported. When a main or interaction effect resulted to be significant ($p\le 0.05$), pairwise comparisons (Bonferroni correction) were reported. Effect size is interpreted as small for 0.01, medium for 0.06, large for 0.14 for $\eta _p^2$ as small for 0.2, moderate for 0.5 and strong 0.8 for Cohen’s d.

## Results

In line with our expectations, we found both *Attractive* and *Repulsive* stimulus encoding to reduce trunk tilt variability and increase the time spent in the dead zone during quiet standing (both Study I and Study II). Also, in line with our expectations, we found an advantage in terms of reduction of sway variability for the *Repulsive* stimulus encoding; however, only during conflict resolution multitasking (Study II). In contrast to our expectations, the *Repulsive* stimulus encoding did not result in an increased cognitive load during working memory multitasking (Study I), while the *Attractive* stimulus encoding did. In contrast, *Attractive* and *Repulsive* stimulus encoding did not differ in terms of cognitive task performance during conflict resolution multitasking.

### Cognitive task performance

#### Study I—working memory

A three-way mixed model ANOVA with the group of vibrotactile feedback instruction (*Attractive*/ *Repulsive*) as between-subject factor and cognitive task (CRT/two-back) and vibrotactile feedback availability (Feedback/no Feedback) as within-subject factors resulted in a significant main effect of Feedback (sphericity assumed: F(1,28)=7.04, p=0.01, $\eta _p^2$=0.20), as well as a Feedback by Group interaction (sphericity assumed: F(1,28)=4.03, p=0.05, $\eta _p^2$=0.13). The main effect of Feedback showed that the corrected response time increased with feedback (mean difference=0.14 s (8%), p=0.05). However, the Feedback by Group interaction indicated that this was only true for the *Attractive* group (mean difference=0.24 s (14%), $p_{adjusted}\le 0.01$, Cohen’s d=1.07). In contrast, for multitasking in the *Repulsive* group, no changes in response times occurred (mean difference=0.03 s (2%), $p_{adjusted}$=0.65) (Fig. C3 left). Moreover, we found a significant main effect of Task (sphericity assumed: F(1,28)=69.78, $p\le 0.001$, $\eta _p^2$=0.71) and interaction of Feedback by Task (sphericity assumed: F(1,28)=10.01, $p\le 0.01$, $\eta _p^2$=0.26). These effects originated from increased corrected response times in the more challenging working memory task (mean difference=0.27 s (12.54%), $p_{adjusted}\le 0.01$, Cohen’s d=0.66), and a stronger task effect when feedback was provided (increase: mean difference=0.86 s (55.45%), $p_{adjusted}\le 0.001$, Cohen’s d=1.79) compared to standing without vibrotactile feedback (increase: mean difference=0.59 s (39%), $p_{adjusted}\le 0.001$, Cohen’s d=1.44).

#### Study II—conflict resolution

The three-way mixed model ANOVA with feedback group (*Attractive*/*Repulsive*) as between-subject factor and the availability of Feedback (Feedback/no Feedback) and cognitive target Congruency (congruent/neutral/incongruent) as within-subject factors for corrected response times revealed a main effect of Feedback availability (sphericity assumed: F(1.00, 33.00) = 51.25, p$\le$0.001, $\eta _p^2$=.61), as well as a significant main effect of Congruency (GG: F(1.05, 34.61)=6.28, p=0.016, $\eta _p^2$=.16). However, the interaction between Feedback availability, Congruency and Group was not significant (GG: F(1.51, 49.93)=1.24, p=0.289, $\eta _p^2$=.036). Pairwise comparisons for the main effect of Feedback availability revealed longer corrected response time when feedback was available compared to when feedback was not available (mean difference=0.11 s (12.92%), p$\le$0.001, Cohen’s d=1.37) (Fig. C3, right). For the main effect of Congruency, pairwise comparisons showed longer corrected response times in the incongruent trials compared to both the congruent and neutral trials (mean difference=0.11 s (12.57%), p=0.054, Cohen’s d=0.49; mean difference=0.10 s (11.30%), p=0.043, Cohen’s d=0.51, respectively).

### Balance control

#### Study I—working memory

The three-way mixed model ANOVA for the variability of trunk tilt angle during multitasking revealed a main effect of Feedback availability (sphericity assumed: F(1,28)=16.63, $p\le 0.001$, $\eta _p^2$=0.37) with lower trunk variability with vibrotactile feedback (mean difference=$0.158^{\circ }$ (21%), $p_{adjusted}\le 0.001$, Cohen’s d=0.87) (Fig. D4, a).

Also, for Time-in-DZ, we found a main effect of Feedback availability (sphericity assumed: F(1,28)=22.37, $p\le 0.001$, $\eta _p^2$=0.44), and a main effect of cognitive Task (sphericity assumed: F(1,28)=4.46, p=0.04, $\eta _p^2$=0.14). These showed the percentage time spent in the dead zone to increase with feedback (mean difference=14.43% (24%), $p_{adjusted}\le 0.001$, Cohen’s d=1.02), and to be lower when the cognitive task involved the more challenging task (mean difference=2.97% (4.36%), $p_{adjusted}$=0.04, Cohen’s d=0.46) (Fig. D5, a).

#### Study II—conflict resolution

Due to a malfunction of the IMU sensor, five participants had to be excluded from the postural performance analysis. Therefore, the following results represent body sway data from 35 participants (*Attractive*: n=18, Repulsive: n=17) only.

A three-way mixed model ANOVA of the variability of trunk tilt angle during the Simon task with feedback group as between-subject factor and feedback availability (Feedback/no-Feedback) and congruency (congruent/neutral/incongruent) as within-subject factors resulted in a main effect of Feedback availability (sphericity assumed: F(1,33)=17.03, p$\le$0.001, $\eta _p^2$=0.34), as well as a marginal interaction between feedback availability by group (sphericity assumed: F(1,33)=3.66, p=0.064, $\eta _p^2$=0.100) and a marginal interaction between feedback availability and congruency (sphericity assumed: F(2,66)=2.92, p=0.061, $\eta _p^2$=0.081). However, there was no three-way interaction effect between feedback, congruency and group (sphericity assumed: F(2,66)=1.07, p=0.35, $\eta _p^2$=0.03). Post-hoc pairwise comparisons for the interaction between the Group and Feedback availability revealed a substantial reduction of sway variability only for the *Repulsive* feedback with a reduction of 40.24% (mean difference=0.66) compared to no-Feedback ($p_{adjusted}$ $\le$0.001, Cohen’s d=1.27) (Fig. D4, b). The significant Feedback by Congruency interaction further showed that sway reduction was apparent across congruency levels (congruent: mean difference=0.42 (28.19%), p$\le$0.001, Cohen’s d=1.14; neutral: mean difference=0.45 (29.80%), Cohen’s d=1.27; incongruent: mean difference=0.49 (32.45%), p$\le$0.001, Cohen’s d=1.27), and that trunk sway variability was lower in incongruent trials compared to congruent trials (mean difference=0.05 (4.67%) p=0.041, Cohen’s d=0.52) when vibrotactile feedback was turned on.

An additional two-way mixed model ANOVA of trunk tilt angle variability during single-task standing and Simon task multitasking without feedback revealed a main effect of Condition (sphericity assumed: F(1,33)=39.43, p$\le 0.001$, $\eta _p^2$=0.54), though no main effect of Group (p=0.390) and no interaction between Group and Condition (p=0.188). Nevertheless, we report post-hoc pairwise comparisons of the interaction, which confirmed no difference between groups in the single-task standing nor in the Simon task multitasking baseline assessments (p=0.70, p=0.26, respectively). However, the pairwise comparisons also revealed that trunk sway variability increased more in the *Repulsive* group (mean difference=0.86 (110.26%), p$\le 0.001$, Cohen’s d=1.03) due to multitasking compared to single-tasking than in the *Attractive* group (mean difference=0.56 (69.14%), Cohen’s d=1.60).

A three-way mixed model ANOVA on the percentage Time-in-DZ during the cognitive conflict resolution task revealed an effect of Feedback availability (sphericity assumed: F(1,33)=104.43, p$\le$0.001, $\eta _p^2$= 0.76), an effect of Congruency (sphericity assumed: F(2,66)=7.28, p=0.001, $\eta _p^2$=0.18), as well as a marginal interaction between the Feedback and Congruency level (sphericity assumed: F(2,66)=2.52, p=0.088, $\eta _p^2$=0.08) and a marginal interaction between Feedback, Congruency and Group (sphericity assumed: F(2,66)=2.89, p=0.063, $\eta _p^2$=0.08). Post-hoc pairwise comparisons of the interaction between Feedback, Congruency and Group demonstrated that the feedback effect with an increased Time-in-DZ compared to no feedback was apparent across both groups and all congruency levels (for all p$\le$0.001) (Fig. D5, b). Finally, the differences between levels of congruency were observable only in the *Repulsive* group with a greater Time-in-DZ in the incongruent compared to congruent trials (mean difference=1.60 (1.98%), p$\le 0.001$, Cohen’s d=1.19) when feedback was on, and a greater Time-in-DZ in the congruent compared to neutral trials (mean difference=1.69 (2.72%), p=0.004, Cohen’s d=0.78) and marginally in the incongruent compared to neutral trials (mean difference=1.47, p=0.06, Cohen’s d=0.73) when feedback was off.

### Subjective evaluation of assistive technology

The analysis of the modified QUEST (Quebec User Evaluation of Satisfaction with Assistive Technology) questionnaire in Study II showed that individuals of both groups were ”quite satisfied” with the vibrotactile feedback with a rating of 4.05 (±0.9) points of 5 points in the *Attractive* group and 4.3 (±7.4) points in the *Repulsive* group. Although overall a multivariate one-way ANOVA did not result in a group difference F(8, 26)=1.24, p=0.317, $\eta _p^2$=0.276, it revealed a difference between Groups in terms of the subscale of Easy-to-use (mean difference=0.68 (19.43%), p=0.040, Cohen’s d=0.63) and marginally for Comfort (mean difference=0.57 (15.32%), p=0.081, Cohen’s d=0.53) (Figure E6). The *Repulsive* group thus experienced the vibrotactile feedback easier to use and more comfortable (mean difference=0.6 points (15.79% higher score, p=0.046). The standardised open question section of the QUEST further showed that 45% of the individuals of the *Attractive* recommended instructing the vibrotactile feedback in a *Repulsive* way might be better. Two individuals described the *Attractive* vibrotactile feedback as “scary” or “confusing,” and five individuals described it as “not intuitive”. In both groups, 9 participants mentioned difficulties with understanding the vibration’s location in relation to the reference sensor’s location and would prefer to receive feedback in the area of the reference sensor location. The other participants abstained from answering the open questions.

## Discussion

Assuming that the interpretation of vibrotactile feedback for the enhancement of balance control involves a diverse range of cognitive processes, we aimed to determine whether *Attractive* or *Repulsive* feedback encoding results in less cognitive costs during cognitive-motor multitasking involving either working memory or cognitive conflict resolution. A further objective was to assess which of the two encodings might be more suitable for the application in daily activities due to a greater balance stabilising effect. Therefore, we investigated in two between-subject studies with healthy young adults how *Attractive* and *Repulsive* encoding of vibrotactile biofeedback affects cognitive and balancing performance while performing either a simple (choice-reaction time task) or more complex (two-back task) auditory working memory task (Study I) or an auditory Simon task (Study II). Kinnaird et al. [47] suggested that *Attractive* feedback may represent a cognitively incongruent stimulus, as it signals an individual to move towards the stimulus. The stimulus, however, represents the boundaries of a stable zone that individuals would intuitively prefer to avoid. Thus, the question arose to what extent the *Attractive* or *Repulsive* encodings show an increased conflict resolution cost.

As expected, and in line with previous works by Haggerty et al. [55] and Lin et al. [41], we observed an increase in response time for the cognitive tasks involving attention and working memory tasks as well as for the cognitive task demanding cognitive conflict resolution when vibrotactile biofeedback was received. However, in contrast to the previous works that investigated the effect of vibrotactile biofeedback (*Repulsive* only) on both a cognitive working memory and balance task performance [41, 55], our Study I showed that the corrected response time indicating increased working memory demands only occurred in the *Attractive* group, and less in the *Repulsive* group (Fig. C3). Further, the increase in corrected response time with feedback occurred only in the more difficult and more complex two-back task, but not in the more simple choice-reaction time task, like it was previously observed in the study by Haggerty et al. [55] and Lin et al. [41]. These differences may be related to the different age groups investigated, for example, due to ageing-related reductions in cognitive multitasking performance. In older adults, as investigated by the other studies [41, 55], cognitive task performance was more strongly affected by additional sensory stimulations (i.e. vibrotactile) than young adults [41, 64]. Our finding that the *Repulsive* encoding imposes lower multitasking costs on the working memory parallels previous studies by Tannert et al. [49] and Kinnaird et al. [47], who reported that the subjective evaluation of the intuitiveness of vibrotactile biofeedback favoured the *Repulsive* feedback encoding. A voluntary balance adjustment in response to a *Repulsive* vibrotactile stimulus comprises an avoidance movement, which seems to enhance cognitive processes [65–67]. Thus, the avoidance movement induced by an instructed *Repulsive* encoding may have facilitated cognitive processes for working memory, explaining the lower cognitive cost in the *Repulsive* group.

The results of our Study II, however, showed that an individual’s performance in the cognitive conflict resolution task did not differ between *Attractive* and *Repulsive* feedback encoding. In both groups, corrected response time was significantly increased in the trials when feedback was available (Feedback) compared to when feedback was not available (no-Feedback). Furthermore, as expected, the interference between balance control and response selection was the greatest in the incongruent trials when conflict resolution was required and Feedback was on. Though, both feedback encodings seem equally demanding in terms of the cognitive conflict resolution required for interpretation and postural response selection.

Regarding control of body balance and in line with previous studies [47, 49], in both of our studies, trunk sway variability decreased, and time spent in the dead zone (feedback inactive) increased with both feedback encodings. The improvement in balance control during multitasking with reduced cognitive task performance in our first study indicates that individuals prioritised balance control over the cognitive task (”posture-first” principle) [17, 37]. Our findings also confirm that working memory storage and updating interfere with balance control in response to an *Attractive* feedback, while this is less the case during balance control in response to the *Repulsive* feedback. In the working memory study, we did not find any differences between *Attractive* and *Repulsive* encoding in terms of balance parameters. In the conflict resolution study, however, we found a slight advantage of *Repulsive* encoding in terms of sway parameters and subjective evaluation. This was reflected by a slightly greater reduction in sway variability. This somewhat greater balance stabilising effect in the *Repulsive* group may be partially explained by the greater increase of sway variability due to the Simon task without vibrotactile feedback in the *Repulsive* group. In analogy to previously reported cognitive facilitation with avoidance behaviour [65–67], another explanation for a stronger reduction of trunk sway with *Repulsive* feedback could be improved efficiency of processing conflicting signals during state estimation and sensory disambiguation. With respect to the subjective evaluations of the feedback, individuals who received *Repulsive* feedback indicated easier use and marginally greater comfort. Moreover, almost half of the individuals of the *Attractive* group recommended using rather *Repulsive* feedback. A *Repulsive* stimulation could be related to the effect of a haptic stimulation, like intermittent poking by passive external light touch or interpersonal light touch [68, 69]. Touching someone lightly on the back and pushing them forward would correspond to a repulsive reaction to the haptic stimulus (away from the stimulus, in the direction of a haptic force vector). Nevertheless, a push is just a transient event, while interpersonal touch is a constant stimulation. Finally, another interesting observation was that when the cognitive task required cognitive conflict resolution during response selection (incongruent trials), trunk sway variability was reduced compared to congruent trials when feedback was on. In a qualitative sense, these observations resemble findings (but described on a different time scale) by Johannsen et al. [16], who found sway variability to be reduced during response selection in incongruent trials of a visual Simon task. They argued that the Simon task may have omitted or delayed any intermittent postural corrections on a short time scale, resulting in lower sway variability. This interference effect was possibly exacerbated by the additional demands of vibrotactile feedback processing. In addition, as the *Repulsive* encoding showed a stronger reduction of sway variability as well as a stronger congruency effect, this may indicate that *Repulsive* feedback encoding shares some overlap with cognitive conflict resolution. Alternatively, the reduced sway variability and increased percentage of time spent in the dead zone in the *Repulsive* group may have also been due to an increased stiffness under increased cognitive load [9]. However, similarly as discussed by Johannsen et al. [16], it is less plausible that the control strategy was switched rapidly between trials since incongruent, congruent and neutral trials appeared in a randomised order within a single block. Finally, in contrast to the working memory tasks in Study I, manual cognitive task responses were faster in Study II (Fig. C3). Thus, the release of sensorimotor balance control from top-down supervision due to the shift of attention away from the body could potentially explain the reduction of sway variability based on the U-shaped model of cognitive-motor interference [17, 20].

Since all participants in our study were healthy young adults, future studies should investigate the effects of *Attractive* and *Repulsive* vibrotactile biofeedback in other target groups, such as in older adults who are generally expected to experience greater cognitive costs during multitasking [41, 55, 64] or also in individuals with balance disorders, such as stroke, dementia, Parkinson’s Disease. Additionally, longer familiarisation trials may be necessary to understand better how vibrotactile biofeedback works to improve overall multitasking performance. As practice previously has been shown to improve both cognitive and sensorimotor task performance [70, 71], multitasking performance, especially in the cognitive task, could be further improved with proper training due to increased automatisation of sensorimotor control. Moreover, as some participants reported difficulties with understanding the location of the feedback (concerning the reference sensor), a follow-up study should compare different locations of the feedback and reference sensors. Finally, follow-up studies are required to investigate to what extent sway variability is reduced more in incongruent trials due to (1) increased stiffness, (2) omitted or delayed intermittent control response, or 3) increased automation of postural control processes by including the assessment of muscle activity and neural processes.

## Conclusion

The *Repulsive* vibrotactile biofeedback encoding was revealed to be less cognitively demanding in terms of working memory than the *Attractive* encoding, which suggests *Attractive* feedback encoding to interfere more with working memory, especially in storage and updating of coordination. However, the increased cognitive load did not negatively affect balance control performance, as individuals of both encoding groups reduced body sway variability and increased the percentage of time spent in the dead zone compared to when feedback was not available. This suggests a posture-first principle to affect cognitive-balance multitasking performance. In contrast, during conflict resolution, a slight advantage of the *Repulsive* encoding became obvious on the postural control level and in subjective evaluations, while Simon task performance did not differ between feedback groups. Due to the advantages of the *Repulsive* feedback encoding in terms of cognitive load while simultaneously solving a working-memory task and in terms of balance control while performing a conflict resolution task, we recommend the *Repulsive* feedback encoding to be the better choice for daily life applications.

## Supplementary Information

Supplementary Material 1.

## Data Availability

The experimental data and the simulation results that support the findings of this study are available in Figshare with the identifier 10.6084/m9.figshare.25459093 (Study I) and 10.6084/m9.figshare.25921270 (Study II).

## Abbreviations

AP: Anterior-posterior
Con: Congruent
CRT: Choice-reaction-time task
DZ: Dead zone
Incon: Incongruent
LISAS: Linear-integrated speed-accuracy score
ML: Medio-lateral
Neutr: Neutral
RMS: Root-mean-square
Time-in-DZ: Percentage of time spent in the dead zone
2back: Two-back task
