# Supplementary material for: Does vibrotactile biofeedback for postural control interfere with cognitive processes?
Source: J Neuroeng Rehabil. 2024 Oct 18;21:184. doi: 10.1186/s12984-024-01476-w (PMC11488272; doi:10.1186/s12984-024-01476-w)
Supplement: Supplementary file 1 — Supplementary Material 1. [file 12984_2024_1476_MOESM1_ESM.pdf]

Appendix A Experimental Procedure

A.1 Study I - Working Memory

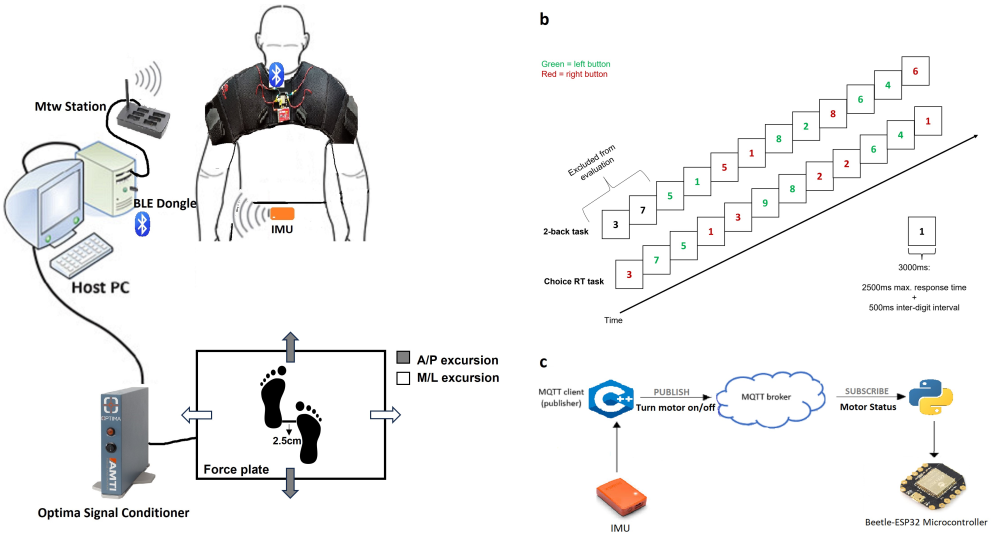

Fig. A1 Experimental setup (a) and auditory cognitive tasks (b) of Study I.

A.2 Study II - Conflict Resolution

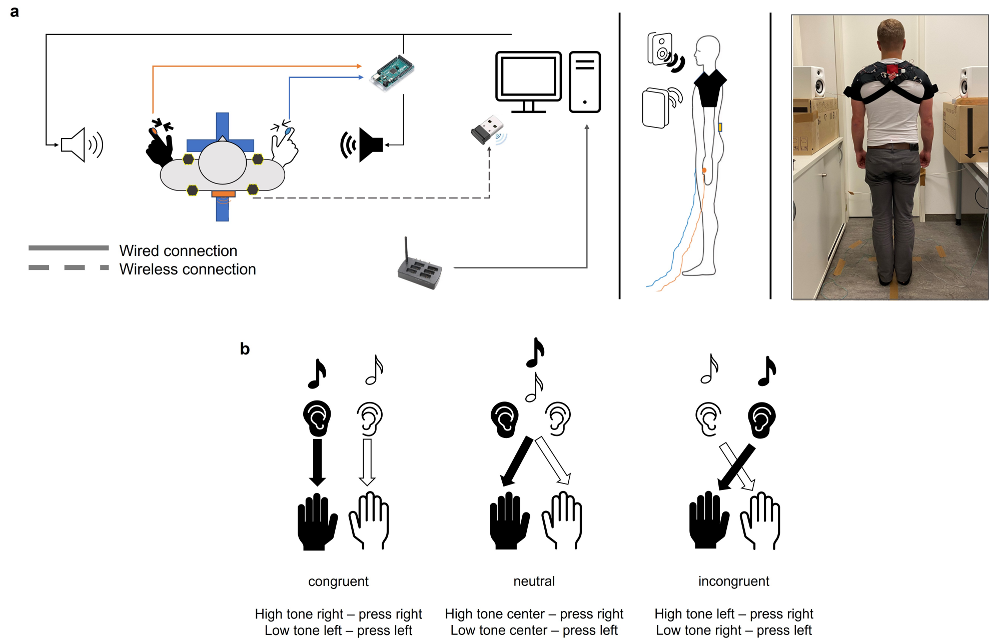

Fig. A2 Experimental setup (a) and auditory Simon task (b) of Study II.

Appendix B Participant Characteristics

The table with participants' characteristics is shown on the next page.

Table B1 Baseline characteristics Study I and II

Study I

Parameter	Attractive group (n=15; 7 males, 8 females)			Repulsive group (n=15; 7 males, 8 females)			
	Whole group (M ± SD)	Males (M ± SD)	Females (M ± SD)	Whole group (M ± SD)	Males (M ± SD)	Females (M ± SD)	Difference of group
Age	26.47 4.21	26.00 5.45	26.67 3.62	26.13 ± 3.56	25.00 ± 1.67	26.00 ± 4.55	p=0.819
Height (m)	1.73 ± 0.09	1.80 ± 0.06	1.67 ± 0.03	1.76 ± 0.11	1.85 ± 0.12	1.70 ± 0.63	p=0.398
Dead zone threshold AP	1.09 ± 0.53	0.99 ± 0.44	1.18 ± 0.62	1.01 ± 0.50	0.85 ± 0.33	1.21 ± 0.61	p=0.72
Dead zone threshold ML	0.47 ± 0.22	0.55 ± 0.20	0.41 ± 0.22	0.41 ± 0.27	0.32 ± 0.11	0.51 ±0.37	p=0.19
Vibrotactile intensity back	51.74 ± 5.49	51.73 ± 5.66	53.10 ± 6.08	52.09 ± 1.30	52.43 ± 0.95	51.79 ± 1.56	p=0.16
Vibrotactile intensity front	48.20 ± 5.17	47.69 ± 5.19	48.90 ± 5.75	48.12 ± 1.22	48.39 ± 0.87	47.89 ± 1.48	p=0.32
Single RMS L5 (°) AP	1.02 ± 0.46	0.92 ± 0.38	1.11 ± 0.52	0.94 ± 0.43	0.80 ± 0.27	1.05 ± 0.52	p=0.42
Single RMS L5 (°) ML	0.44 ± 0.19	0.50 ± 0.17	0.38 ± 0.20	0.38 ± 0.24	0.30 ± 0.09	0.46 ± 0.30	p=0.19

Study II

Parameter	Attractive group (n=18; 6 males, 12 females)			Repulsive group (n=17; 6 males, 11 females)			
	Whole group (M ± SD)	Males (M ± SD)	Females (M ± SD)	Whole group (M ± SD)	Males (M ± SD)	Females (M ± SD)	Difference of group
Age	28.17 ± 3.84	26.5 ± 3.56	29.00 ± 3.83	28.71± 4.96	31.33 ± 5.2	27.27 ± 4.41 p=0.720	
Height (m)	169.67 ± 7.52	177.83 ± 3.87	165.58 ± 5.09	167.59 ± 10.88	177.667 ± 10.1	162.09 ± 6.65	p=0.513
Dead zone threshold AP	0.63 ± 0.38	0.72 ± 0.62	0.58 ± 0.19	0.52 ± 0.14	0.58 ± 0.14	0.47 ± 0.13	p=0.269
Dead zone threshold ML	1.33 ± 0.351	1.28 ± 0.37	1.35 ± 0.35	1.53 ± 0.55	1.3 ± 0.32	1.65 ± 0.62	p=0.204
Vibrotactile intensity back	57.22 ± 6.236	57.50 ± 5.24	57.08 ± 6.89	59.41 ± 7.88	55.0 ± 5.48	61.81 ±8.15	p=0.367
Vibrotactile intensity front	52.22 ± 6.691	51.67 ± 5.16	52.5 ± 7.53	53.53 ± 7.66	49.167 ± 6.646	55.91 ± 7.35	p=0.594
Single RMS L5 (°) Total Tilt	0.81 ± 0.24	0.83 ± 0.12	0.81 ± 0.29	0.78 ± 0.22	0.68 ± 0.20	0.835 ± 0.22	p=0.695

Appendix C Result Figures - Cognitive Performance

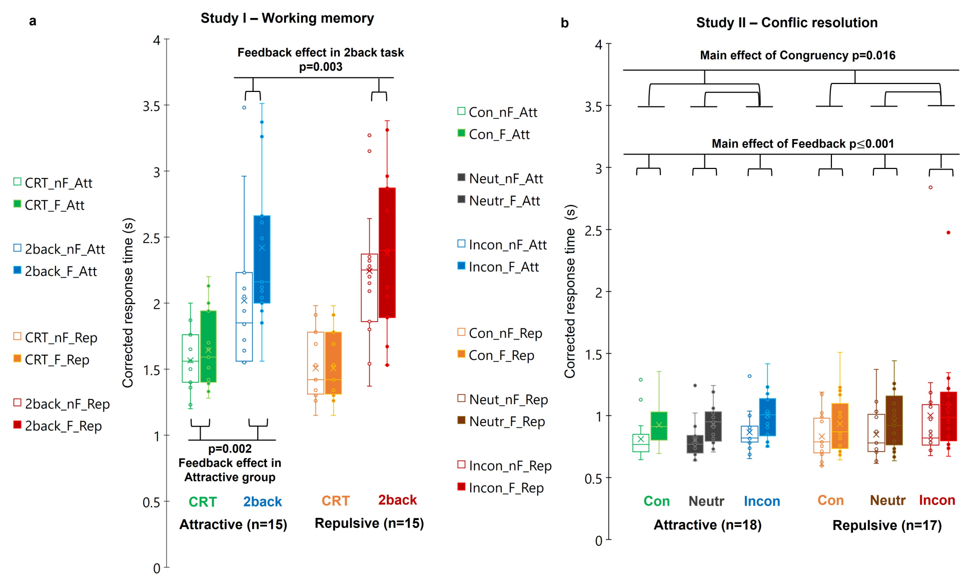

Fig. C3 Secondary task performance for working memory tasks (choice-reaction time task (CRT) and two-back task (2back) in Study I, left) and the different levels of congruency (congruent (Con), neutral (Neutr), incongruent (Incon)) during the auditory conflict resolution task, the Simon task (Study II, right). Box-plots presented for the corrected response time in seconds based on the linear-integrated speed-accuracy trade-off score (LISAS) for *Attractive* (green, blue, grey) and *Repulsive* (orange, red, kaki) encoding. Significant and marginal pairwise comparisons of the Feedback x Group interaction and Feedback x Task interaction for Study I, as well as the main effect of Feedback and the main effect of Congruency are marked with p-values.

Appendix D Result Figures - Balance Performance

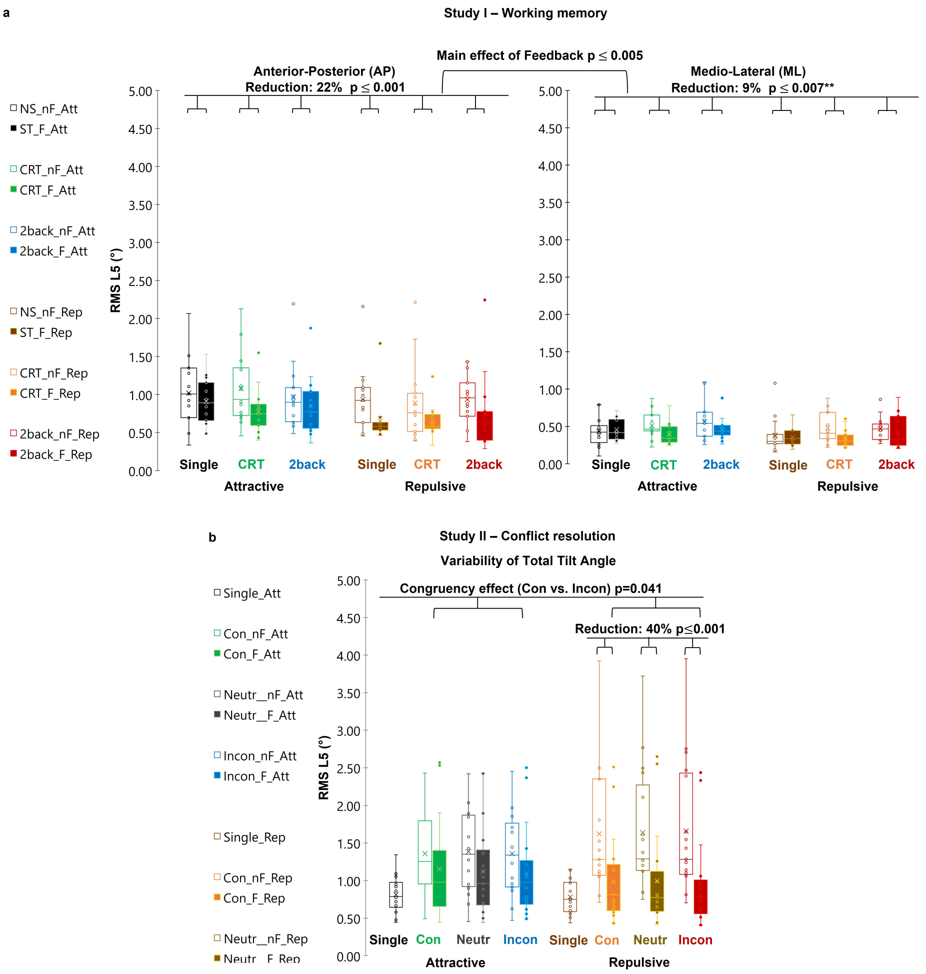

Fig. D4 Box-plots of RMS of L5 tilt angle (°) for single-task standing and multitasking with working memory tasks (choice-reaction time task (CRT) and two-back task (2back) in Study I, **a**) and with different levels of congruency (congruent (Con), neutral (Neutr), incongruent (Incon)) during secondary auditory conflict resolution task, the Simon task (Study II, **b**) for *Attractive* (green, blue, grey, black) and *Repulsive* (orange, red, kaki, brown) encodings. While in Study I we differentiated in anterior-posterior (AP) direction (left) and medio-lateral (ML) direction (right), in Study II, we analysed the total tilt, combining AP and ML. The main effect of Feedback for Study I and significant and marginal pairwise comparisons of the Feedback x Group and Feedback x Congruency interactions for Study II are marked with p-values.

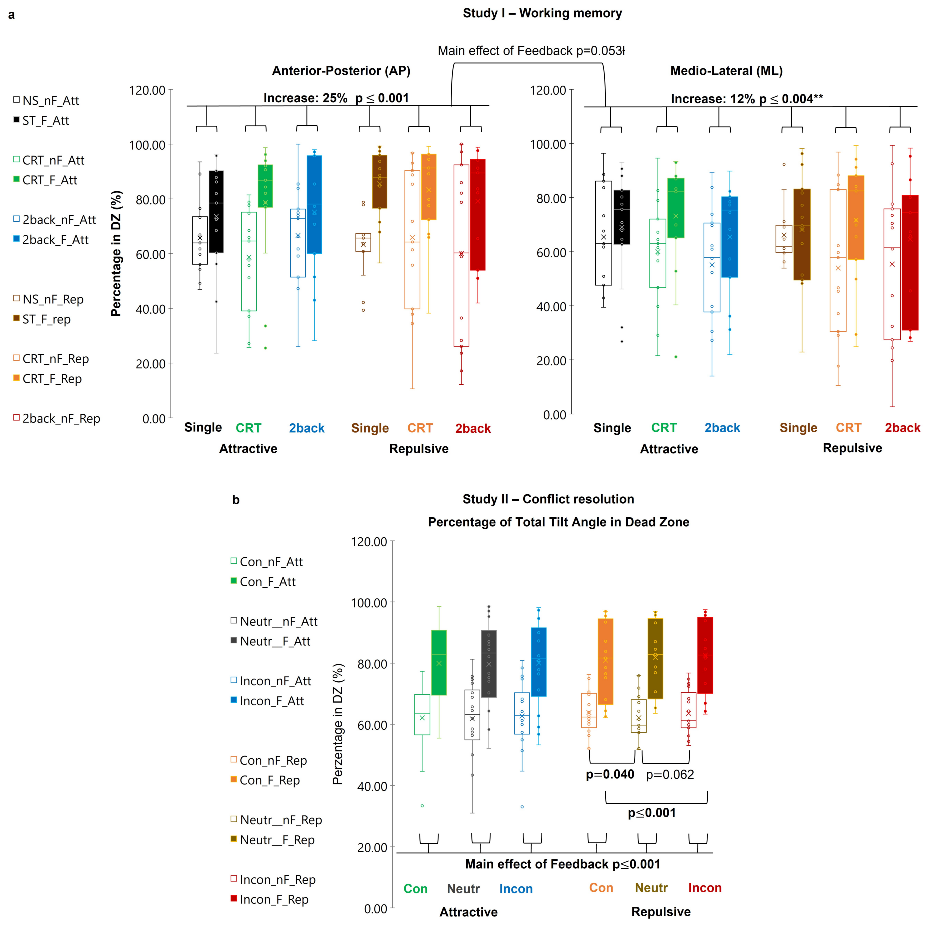

Fig. D5 Study I: Box-plots of the percentage of time spent in the dead zone (DZ) for single-task standing and multitasking with working memory tasks (choice-reaction time task (CRT) and two-back task (2back) in Study I, **a**) and with different levels of congruency (congruent (Con), neutral (Neutr), incongruent (Incon)) during secondary auditory conflict resolution task, the Simon task (Study II, **b**) for *Attractive* (green, blue, grey, black) and *Repulsive* (orange, red, kaki, brown) encodings. While Study I differentiated in anterior-posterior (AP) direction (left) and medio-lateral (ML) direction (right), in Study II, we analysed the total tilt, combining AP and ML. The main effect of Feedback for Study I and significant and marginal pairwise comparisons of the Feedback x Congruency x Group interaction for Study II marked with p-values.

Appendix E Result Figures - Subjective Evaluation

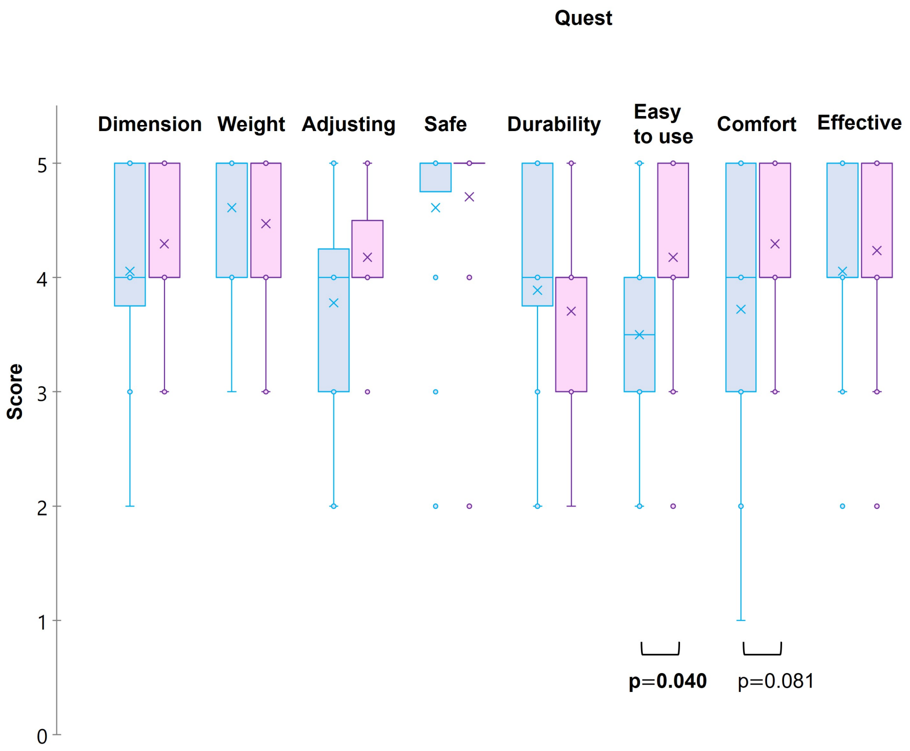

Fig. E6 Study II: Box-plots of QUEST subitems for *Attractive* (blue) and *Repulsive* groups (purple). Marginal and significant pairwise comparisons of the Group x Subitems interaction are marked with p-values.
